# Effect of Prescriber Notifications of Patient’s Fatal Overdose on Opioid Prescribing at 4 to 12 Months: A Randomized Clinical Trial

**DOI:** 10.1001/jamanetworkopen.2022.49877

**Published:** 2023-01-06

**Authors:** Jason N. Doctor, Emily Stewart, Roneet Lev, Jonathan Lucas, Tara Knight, Andy Nguyen, Michael Menchine

**Affiliations:** 1 Leonard D. Schaeffer Center for Health Policy and Economics, University of Southern California, Los Angeles; 2 Department of Pharmaceutical and Health Economics, School of Pharmacy, University of Southern California, Los Angeles; 3 Sol Price School of Public Policy, University of Southern California, Los Angeles; 4 Emergency Department, Scripps Mercy Hospital San Diego, San Diego, California; 5 Department of Medical Examiner-Coroner, County of Los Angeles, Los Angeles, California; 6 Global Blood Therapeutics, South San Francisco, California; 7 Department of Emergency Medicine, University of Southern California, Los Angeles

## Abstract

This randomized clinical trial evaluates the effect of prescriber notifications of a patient’s fatal overdose on opioid prescribing, including decreases in morphine milligram equivalents, new patients taking opioids, and patients taking a high dose, at 4 to 12 months after notification.

## Introduction

Doctor et al^1^ sought to establish whether clinicians notified by their county’s medical examiner of their patient’s overdose from a schedule II to IV drug were more likely than clinicians who were not notified to reduce opioid prescribing. The trial involved 167 decedents who received prescriptions from 826 clinicians in San Diego County, California, from July 2015 through June 2016.^1^ The results showed a 9.7% decrease in prescriptions filled for morphine milligram equivalents (MME) up to 3 months after letter receipt.^1^ In addition, the trial found a decrease in new patients and patients taking a high dose of opioids in the panels of those clinicians receiving the letter.^1^ This analysis using data from a previous randomized clinical trial evaluates whether decreases in MME, new patients taking opioids, and patients taking a high dose persisted up to 1 year after clinicians received a letter about a patient’s fatal overdose.

## Methods

This randomized clinical trial was preregistered at ClinicalTrials.gov (NCT02790476). Details of the intervention, sample, design, and analysis are found in Doctor et al.^1^ The study protocol is shown in Supplement 1. The University of Southern California institutional review board approved this study and waived informed consent because (1) the research was performed by local (eg, county) government officials and was designed to study an intervention of public benefit, and (2) the research could not practicably be performed without the waiver. We followed the Consolidated Standards of Reporting Trials (CONSORT) reporting guidelines.

Clinicians were included if they had prescribed an opioid within the 12 months before a patient’s death from July 1, 2015, to June 30, 2016. We analyzed 12 months of data before and after the intervention. We evaluated the change in total weekly MMEs dispensed using a mixed-effects regression with interactions between conditions, at 1 to 3 months and 4 to 12 months after the intervention. The model was left-censored to account for weeks with no prescribing. We used logistic regression to analyze new starts on opioids and high-dose prescriptions of at least 50 and 90 MME. We report model-adjusted 5% trimmed means and bootstrapped 95% CIs.^2^ Using a *t* test, we also evaluated whether there was any degradation in treatment effect by coefficients at the 2 time points.^2^ Two-sided *P* < .05 was considered statistically significant. We completed the analysis using Stata statistical software version 16 (StataCorp) and SAS statistical software version 9.4 (SAS Institute). Data were analyzed from March to November 2022.

## Results

We excluded clinicians who were no longer practicing at follow-up, eliminating 19 prescribers and 1 corresponding decedent (Figure). The final sample contained 166 decedents and 809 clinicians. Prescribers in the original sampe were predominantly medical doctors (592 individuals [64.41%]), followed by physician assistants (92 individuals [11.14%]), and doctors of osteopathy (59 individuals [7.14%]). The primary cause of death was attributable to an opioid prescription (94 deaths [56.29%]), rather than the combined effects of an illicit drug (42 deaths [26.15%]) or alcohol (23 deaths [13.77%]). There were 223 399 opioid prescriptions dispensed 4 to 12 months after the intervention.^1^ Total weekly MMEs dispensed decreased 7.1% (95% CI, 0.37% to 13.79%; *P* = .04) more in the letter intervention group than in the control group 4 to 12 months after the intervention. The Table shows the absolute change in MMEs at 1 to 3 and 4 to 12 months after the intervention. New patients taking opioids decreased by 2.02 percentage points (95% CI, 0.79 to 3.25 percentage points; *P* = .001) more 4 to 12 months after intervention among intervention than control prescribers, respectively. There was no difference between effects at 1 to 3 and 4 to 12 months for total MME change (β = −0.02; 95% CI, −0.11 to 0.08; *P* = .71) or new patients (β = 0.01; 95% CI, −0.10 to 0.13; *P* = .83). The number of prescriptions that were at least 50 MME (β = −0.06; 95% CI, −0.14 to 0.02; *P* = .20) or 90 MME (β = 0.06; 95% CI, −0.05 to 0.16; *P* = .27) did not decrease at 4 to 12 months.

**Figure.**
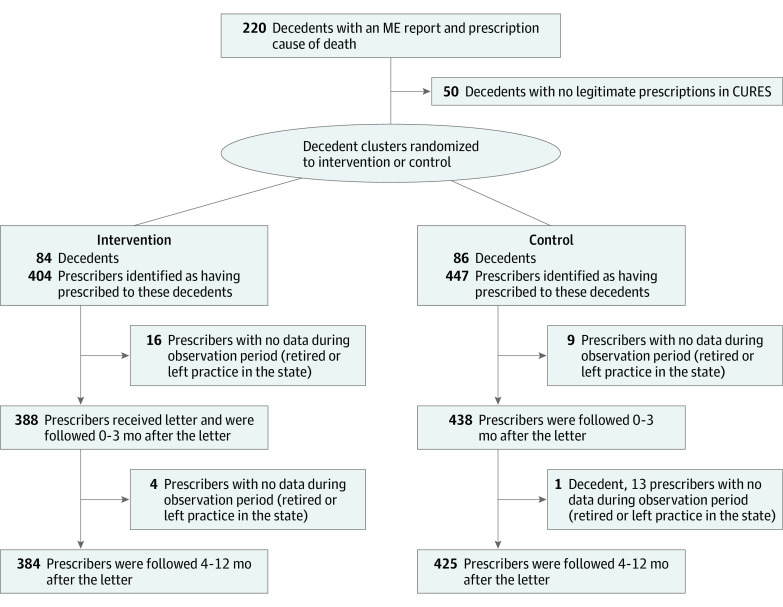
Study Flowchart CURES indicates Controlled Substance Utilization Review and Evaluation System; ME, medical examiner.

**Table.** Adjusted Per-Prescriber Weekly MMEs After Intervention

Parameter	MMEs, mean (95% CI)	
	Letter (n = 385 prescribers)	Control (n = 424 prescribers)
1-3 mo^a^		
Preintervention	328.43 (320.25 to 336.60)	329.14 (321.93 to 336.35)
Postintervention	263.70 (257.17 to 270.24)	288.97 (282.39 to 295.56)
4-12 mo^b^		
Preintervention	328.43 (320.25 to 336.60)	329.14 (321.93 to 336.35)
Postintervention	131.54 (128.29 to 134.79)	141.50 (138.20 to 144.79)

Abbreviation: MME, morphine milligram equivalent.

^a^ The difference in differences was –24.56 MMEs (95% CI, –34.19 to –14.71 MMEs).

^b^ The difference in differences was –9.24 MMEs (95% CI, –14.00 to –4.39 MMEs).

## Discussion

This randomized clinical trial found that opioid prescribing continued to decrease well after receipt of a letter notifying the clinician of a fatal overdose. Awareness of being observed, an injunction from authority, and the availability of harm may explain this effect. The intervention was delivered at a low cost. This study did not address the alarming acceleration of fatal overdoses by illicit fentanyl. Considering the changing pandemic-related causes of death, future research should assess patient-reported outcomes and determine whether such letters could encourage medication-assisted therapy initiation.

## Notes

Supplement 1.
Trial Protocol

Supplement 2.
Data Sharing Statement
