# Supplementary material for: Effect of Prescriber Notifications of Patient’s Fatal Overdose on Opioid Prescribing at 4 to 12 Months: A Randomized Clinical Trial
Source: JAMA Netw Open. 2023 Jan 6;6(1):e2249877. doi: 10.1001/jamanetworkopen.2022.49877 (PMC9856831; doi:10.1001/jamanetworkopen.2022.49877)
Supplement: Supplement 1. — Trial Protocol [file jamanetwopen-e2249877-s001.pdf]

STUDY PROTOCOL

Behavioral insights to encourage judicious prescribing of opioids: A cluster randomized trial on prescribing behaviors for scheduled drugs

Principal Investigator:

Jason Doctor, Ph.D., Professor, University of Southern California

Supported by:

The California Health Care Foundation

Date: February 23, 2016

TABLE OF CONTENTS

Page

STUDY TEAM ROSTER **1**

PARTICIPATING STUDY SITES **1**

PRÉCIS **2**

1. Study objectives **3**

1.1 Primary Objective 3

1.2 Secondary Objectives 3

2. BACKGROUND AND RATIONALE **3**

2.1 Background on Condition, Disease, or Other Primary Study Focus 3

2.2 Study Rationale .... **Error! Bookmark not defined.**

3. STUDY DESIGN **4**

4. SELECTION AND ENROLLMENT OF PARTICIPANTS **5**

4.1 Inclusion Criteria 5

4.2 Exclusion Criteria 5

4.3 Study Enrollment Procedures 5

5. STUDY INTERVENTIONS **5**

5.1 Interventions, Administration, and Duration 5

5.2 Handling of Study Interventions 6

5.3 Adherence Assessment 6

6. STUDY PROCEDURES **6**

6.1 Schedule of Evaluations .... **Error! Bookmark not defined.**

6.2 Description of Evaluations .... **Error! Bookmark not defined.**

6.2.1 Screening Evaluation 7

6.2.2 Enrollment, Baseline, and/or Randomization 7

6.2.3 Follow-up Visits 7

6.2.4 Completion/Final Evaluation 8

7. SAFETY ASSESSMENTS **8**

7.1 Specification of Safety Parameters 8
7.2 Methods and Timing for Assessing, Recording, and Analyzing Safety Parameters 8
7.3 Adverse Events and Serious Adverse Events 8
7.4 Reporting Procedures 9
7.5 Follow-up for Adverse Events 9
7.6 Safety Monitoring 10

8. INTERVENTION DISCONTINUATION 10

9. STATISTICAL CONSIDERATIONS 10

9.1 General Design Issues 10
9.2 Sample Size and Randomization 10
9.2.1 Treatment Assignment Procedures 11
9.3 Interim analyses and Stopping Rules 11
9.4 Outcomes 12
9.4.1 Primary outcome 12
9.4.2 Secondary outcomes 12
9.5 Data Analyses 12

10. DATA COLLECTION AND QUALITY ASSURANCE 13

10.1 Data Collection Forms 13
10.2 Data Management 13
10.3 Quality Assurance 14
10.3.1 Training 14
10.3.2 Quality Control Committee 14
10.3.3 Metrics 14
10.3.4 Protocol Deviations 14
10.3.5 Monitoring 14

11. PARTICIPANT RIGHTS AND CONFIDENTIALITY 15

11.1 Institutional Review Board (IRB) Review 15
11.2 Informed Consent Forms 15
11.3 Participant Confidentiality 15
11.4 Study Discontinuation 15

12. COMMITTEES 15

13. PUBLICATION OF RESEARCH FINDINGS 15

14. REFERENCES..16

15. SUPPLEMENTS/APPENDICES 16

I. Procedures Schedule

II. Informed Consent Form Template

Not applicable - we will request a waiver of consent.

III. Other *(add as many appendices as necessary)*

STUDY TEAM ROSTER

Jason N. Doctor, Ph.D. (Principal Investigator), Leonard D. Schaeffer Center for Health Policy and Economics, University of Southern California, 635 Downey Way, Los Angeles, CA 90089-7273, Office: 213.821.8142, Fax: 213.740.3460, jdoctor@usc.edu

Roneet Lev, MD (Co-Investigator), Scripps Mercy Hospital Emergency Department and San Diego County Rx Drug Abuse Medical Task Force, levreviews@gmail.com

Jonathan Lucas, MD (Co-Investigator), San Diego County Medical Examiner's Office, 5570 Overland Ave Suite 101, San Diego, CA 92123-1215, Jonathan.Lucas@sdcounty.ca.gov

Tara K. Knight, Ph.D. (Project Manager), Leonard D. Schaeffer Center for Health Policy and Economics, University of Southern California, 635 Downey Way, Los Angeles, CA 90089-7273, Office: 213.821.7943, Fax: 213.740.3460, knight@usc.edu

Michael Menchine, MD MPH (Co-Investigator), Leonard D. Schaeffer Center for Health Policy and Economics, University of Southern California, 635 Downey Way, Los Angeles, CA 90089-7273, Office: 213.821.7943, Fax: 213.740.3460, mmenchin@gmail.com

PARTICIPATING STUDY SITES

San Diego County Medical Examiner's Office. Jonathan Lucas, MD, Chief Deputy Medical Examiner, 5570 Overland Ave Suite 101, San Diego, CA 92123-1215, (PHONE/FAX), Jonathan.Lucas@sdcounty.ca.gov

PRÉCIS.

Study Title: Behavioral insights to encourage judicious prescribing of opioids

Objectives

In collaboration with the San Diego Medical Examiner's Office and the State of California's controlled Substance Utilization Review and Evaluation System (CURES), we propose to review opioid poisonings over the past 12 months and will send letters to prescribers in San Diego County when one of their prescriptions was filled by a patient who died of an opioid poisoning. The letters will be non-judgmental and factual, explaining that a patient of theirs who was being treated with prescription narcotics died of an opioid poisoning. The letter will also encourage judicious prescribing including use of the CURES system before prescribing. We will evaluate physician prescribing practices over 24 months (12 months pre- and 12 months post-letter) using data from the CURES database. Our hypothesis is that letters will make the risk of opioids more cognitively available and that physicians will respond by prescribing them more carefully. This will result in fewer deaths due to misuse and more frequent use of the CURES system.

Design and Outcomes

We will conduct a cluster randomized trial of an informative intervention targeting the over-prescription of scheduled drugs with patient death as the unit of randomization.

The follow outcomes will be analyzed:

1) Fewer opioid prescriptions written among clinicians receiving the letter, compared to controls;
2) Lower cumulative Morphine Equivalent Dose (cMED) among clinicians receiving the letter (as indicated by dosing on prescriptions filled);
3) More frequent use of CURES among clinicians receiving a letter;
4) Fewer opioid prescriptions for ≥ 100 milligram morphine equivalent (MME)
5) Fewer benzodiazepine co-prescriptions

In the main analysis, we will compare a 12-month baseline period to a 12-month post-treatment follow-up period.

Interventions and Duration

The intervention will be a one-time treatment for all participants, with a three-month and a one year follow-up period to measure the persistence of the effects after the intervention.

The following intervention will be analyzed:

A courtesy communication informing prescribers of the death of a former patient where prescription drug overdose was the primary cause or contributed to the cause of the death. The letter will invite prescribers to review materials regarding the clinical guidelines from the Center of Disease Control, the California Department of Public Health, and the San Diego County Prescription Drug Abuse Medical Task Force for a more prudent prescription of those drugs.

Sample Size and Population

Our sample will consist of a heterogeneous group of clinicians and allied health professionals with scheduled drug prescribing privileges practicing in San Diego County who prescribed a Schedule II-IV drug (i.e., drugs as defined by the United States Controlled Substances Act as having an abuse potential, but also a currently accepted medical use) to a person who died as a result of a Schedule II-IV accidental drug death.

1. STUDY OBJECTIVES

1.1 Primary Objective

Our primary objective is to analyze prescribers in the treatment group's milligram morphine equivalent dose (mg MED) compared to the control participants before and after the intervention.

1.2 Secondary Objectives

Our secondary objective is to observe frequency of CURES use and the number of "new starts".

2. BACKGROUND AND RATIONALE

2.1 Background on Condition, Disease, or Other Primary Study Focus

Much of the increase in opioid prescribing rates from 1999 until the past decade has been driven by an increase in the use of prescription opioids to treat non-cancer pain (Guy et al. 2017; Boudreau et al. 2009). Although opioids carry significant risks of overdose and addiction, they are no more effective for treating chronic non-cancerous pain over a one year period than non-opioid alternatives (Krebs et al. 2018). The greater availability of prescription opioids has been accompanied by an alarming rise in the negative consequences related to opioid use. In 2017, there were 17,029 prescription opioid overdose deaths in the US (Scholl 2019). The costs of prescription opioid adverse outcomes are staggering. Aggregate costs for prescription opioid harms are estimated at over $78.5 billion (in 2013 USD) and 25% of the aggregate economic burden is publicly funded (i.e., Medicaid, Medicare, and veterans' programs) (Florence et al. 2016).

In 2016, the Centers for Disease Control and Prevention (CDC) issued the "CDC Guideline for Prescribing Opioids for Chronic Pain" which encourages the use of alternatives to opioids and other practices that minimize harm to patients (Dowell et al. 2016). Despite the introduction of this guideline, primary care clinicians, who prescribe 45% of all opioid prescriptions in the US, report challenges in following these recommendations (Kroenke et al. 2019). The dynamics of opioid use make following guidelines difficult. Since opioid analgesia from a given dose declines with chronic use due to opioid tolerance, doses increase and the chance of harm grows. Over time, the primary benefit of opioids for many patients becomes the avoidance of withdrawal. As patients become dependent on opioids, they may misconstrue the treatment of interdose withdrawal hyperalgesia as ongoing effectiveness and they may become reluctant to stop opioids

(Juurlink 2017). More cautious opioid prescribing (including fewer new starts, avoidance of high doses, and slow, collaborative tapers for those already on high dose long-term therapy) can improve the balance of benefits and harms for patients with chronic pain. To embrace more cautious prescribing, a clinician may need to be informed that opioid risks are present and relevant to his/her own patients.

2.2 Study Rationale

Prescriber's invited to join efforts to exercise more careful use of opioids may be more likely to do so after they have been made aware of an opioid death in their practice. They may also be more likely to dedicate themselves to amending their prescribing practices, committing to become an "aide in practice" and identify careful use of opioids with their self-image in the future. Many physicians underestimate the risk of opioids in their own practice, and currently, there is no mechanism for medical examiners to identify prescribing physicians, nor for physicians to be alerted about deaths, despite substantial interest from the CDC on solving this problem. One promising strategy for changing physician behavior is through use of "nudges". The term "nudge" identifies a set of social and cognitive devices that persuade decisions in subtle ways while preserving choice. When prescribing narcotics, physicians may not have recent experiences that come to mind as to why these drugs are dangerous, making risks seem remote. They may also not consult data on a patient's other narcotic prescriptions in their State's pharmaceutical drug monitoring program. One useful nudge capitalizes on a finding called "Availability". Availability is the notion that people rely upon knowledge that is salient, recent and readily available to them to evaluate risks and make decisions. If a physician issued a prescription for a narcotic that resulted in a recent opioid poisoning, providing that physician with this feedback may make the physician more likely to consult a pharmaceutical drug monitoring program database and also prescribe more judiciously.

3. STUDY DESIGN

The trial is a cluster-randomized controlled trial with prescriber as the unit of randomization. The primary aim is to test the ability of the informative intervention to reduce the number of Scheduled drug prescriptions. We will randomize prescribers in a 1:1 design where half will be in the control group while the other half receives the intervention. All physicians involved in prescription drug over-dosage related deaths in the 12-month baseline period will be randomized to receive a letter regarding that death as well as guidelines for future consideration regarding prescribing practices. The intervention will be followed by a one year follow-up period to measure persistence of effects. Data from the Controlled Substance Utilization Review and Evaluation System 2.0, which is California's prescription drug monitoring program, will be extracted and transferred to the Data Coordinating Center on a weekly basis.

4. SELECTION AND ENROLLMENT OF PARTICIPANTS

4.1 Inclusion Criteria

The subjects involved in this trial are clinicians will be from those who prescribe Scheduled drugs and practice in the County of San Diego. Clinicians must meet the following inclusion criteria to be selected to participate in the study: 1) have given at least one prescription for any sort of opioid medication to patients and 2) prescribed to a patient who have deceased within the 12-month baseline period where prescription drug overdose was the primary cause or contributed to the cause of death.

4.2 Exclusion Criteria

Clinicians not meeting inclusion criteria above will be excluded from the study.

4.3 Study Enrollment Procedures

Participants will not be enrolled; we will intervene on all clinicians and allied health professionals with prescribing privileges and at least one prescription drug death as part of a public health program to encourage safe prescribing in San Diego County. Informed consent of patients and clinicians is waived under HHS regulations at 45 CFR 46.116(c), as the study is evaluating a County public service safe prescribing program. Deaths where prescription drugs are the primary or contributing cause will be cataloged by the medical examiner between the period of July 1st, 2015 and June 30th, 2016. As part of the County safe prescribing program, prescribers to those deaths are identified in the CURES system. All California pharmacies and clinics that dispense controlled substances must submit reports to CURES on a weekly basis. In consultation with the program manager of CURES, a letter notifying CURES administrators that prescriptions from these clinicians would be evaluated prospectively after the safe prescribing letters were sent was submitted to the CURES system.

5. STUDY INTERVENTIONS

5.1 Interventions, Administration, and Duration

A letter signed by the Chief Deputy Medical Examiner of San Diego County will notify prescribers of the death in their practice. The letter will outline the annual number and types of prescription drug deaths seen by the medical examiner, discussed the value of and way to access the State's prescription drug monitoring program, and discussed five CDC guideline-recommended safe prescribing strategies: 1) Avoid co-prescribing of opioids with benzodiazepines, 2) prescribe minimal dose necessary for acute pain, 3) consider slow tapers with pauses to below 50 MME per day, 4) avoid prescriptions lasting greater than 3-months for

pain, and 5) prescribe naloxone in conjunction with opioids for patients taking ≥ 50 MME per day.

5.2 Handling of Study Interventions

Letters will be carefully edited by an advisory group to ensure tone is supportive and its recommendations consistent with CDC guidelines. Letters will be generated, signed by the San Diego Medical Examiner and posted in the U.S. mail. The pre-intervention baseline period will be 12 months in length with a one year follow-up period to measure the persistence of effects after the intervention.

5.3 Adherence Assessment

Adherence is compulsory as prescription fills are automatically processed through pharmacies in the State and sent to the State's prescription drug monitoring program.

6. STUDY PROCEDURES

6.1 Schedule of Evaluations

Assessment	Baseline	Follow-up: 4 Months	Follow-up: 12 months
Schedule drug prescriptions	x	x	x
mg MED/MME	x	x	x
Physician specialty	x		
Board certification	x		
Years licensed in state	x		
Prescription drug related death for prescriber	x		
Inclusion/Exclusion Criteria	x		
Enrollment/Randomization	x		
Consent Waiver	x	x	x

6.1.1 **Screening Evaluation**

All clinicians whom meet the inclusion criteria will be eligible for the trial. Eligibility will be based on historical data from the CURES database.

Consenting Procedure

Not applicable. We will be requesting a waiver of consent under HHS regulations at 45 CFR 46.116(c).

6.1.2 **Enrollment, Baseline, and/or Randomization**

Enrollment

Participants will not be enrolled; we will intervene on all clinicians and allied health professionals with prescribing privileges and at least one prescription drug death as part of a public health program to encourage safe prescribing in San Diego County. Informed consent of patients and clinicians is waived under HHS regulations at 45 CFR 46.116(c), as the study is evaluating a County public service safe prescribing program.

Baseline Assessments

For the baseline assessment, the study team will evaluate physician prescribing practices in the 12 months pre- intervention using data from the CURES database.

Randomization

For randomization, six decedent lists were generated from the crossed strata levels. Using random.org's sequence generator,(*16*) true random integer sequences derived from atmospheric noise determined decedent order in each list. For each ordered list, prescribers to decedents in the first half were those who received the intervention.

6.1.3 **Follow-up Visits**

The follow-up observation period includes evaluating physician prescribing practices in the 13 months following letter receipt.

6.1.4 **Completion/Final Evaluation**

A participant is considered to have completed the study if he or she has completed the baseline assessment, received a letter, and been observed for >13 months following letter receipt. The end of the study is defined as completion of the >13-month data observation period following receipt of letter.

7. SAFETY ASSESSMENTS

7.1 Specification of Safety Parameters

Although we will be unable to determine if there are hospitalizations or ED visits for opioid withdrawal in patients of clinicians in the intervention group, we will monitor this potential safety issue by comparing clinicians who received a letter to controls to determine if there are any dramatic reductions (>20% from baseline) in opioid prescribing in the post-intervention period as a result of the letter. The U.S. does not have an integrated healthcare system with complete data on hospitalizations for all patients to evaluate induced withdrawal. However, it is possible for us to evaluate if providers induce withdrawal in patients by exhibiting high rates of MME reductions.

7.2 Methods and Timing for Assessing, Recording, and Analyzing Safety Parameters

We will assess safety throughout the course of the intervention period and follow-up period.

7.3. Adverse Events and Serious Adverse Events

At any time, clinicians can report an adverse event or unanticipated problem potentially related to the letters to the medical examiner's office or the County or City public health officer who co-signs the letter and provides contact information.

We do not expect there to be adverse events directly influenced by the clinical guidance being delivered in this study. All study interventions encourage clinicians to follow well-established national guideline recommendations and known best practices. While the expectedness of adverse events is very low, we will investigate each and every numerator case identified in all safety measures described above. For cases identified by the safety monitoring measures, we will perform manual physician chart review to examine the clinical circumstances and to make a judgment (1) the expectedness of the event [unexpected, expected], (2) the likelihood that the safety event was study related [not related/possibly related/definitely related] and (3) judge the event's severity [abnormal clinical finding without symptoms/symptoms requiring clinical

intervention/short term disability or hospitalization/death AND separately define the severity as mild, moderate, or severe].

These will be conducted only by authorized study personnel. Study personnel will interview clinicians treating patients when needed to obtain additional information. Each case identified will have a case report form with these variables and will be signed and dated by study staff completing the form. These forms will be stored in a locked office. Each adverse event will be given an identification number. If study personnel believe that a patient that experienced an adverse event would benefit from seeing or communicating with their clinician who previously received a letter, the PI will within 2 business days reach out to this clinician advising them to contact the patient as soon as possible.

7.4 Reporting Procedures

The seriousness of the adverse event will be documented on the case report form. We will categorize all of the following as serious adverse events: patient death, life-threatening event, hospitalization-initial or prolonged, disability/incapacity, and events that required intervention to prevent permanent impairment. The clinician reviewing the event will determine the seriousness of the event. If it is an event other than those listed above that the reviewing clinician feels is an 'other' serious event, it will be discussed with another clinical study team member to reach consensus. All details will be documented on a case report form.

We will within a business day report any clinician reported adverse events, safety analysis, or unanticipated problems to the USC IRB. Our report will include appropriate identifying information for the study, a detailed description of the adverse event, and a description of any changes to the protocol or other corrective actions that have been taken or are proposed. If an adverse event occurs, we will review relevant clinical decision support and ensure others are not at a greater risk of harm than was previously known or recognized. Clinician participants are only receiving a letter informing them of a recent patient death due to opioid overdose. Additional patient deaths related to this study are not expected. However, should we identify a patient death in safety measures described above we will report the death to the USC IRB within 24 hours of our knowledge of the death.

7.5 Follow-up for Adverse Events

We will within a business day report any clinician reported adverse events, safety analysis, or unanticipated problems to the USC IRB. Our report will include appropriate identifying information for the study, a detailed description of the adverse event, and a description of any changes to the protocol or other corrective actions that have been taken or are proposed. If an adverse event occurs, we will review relevant clinical decision support and ensure others are not at a greater risk of harm than was previously known or recognized.

7.6 Safety Monitoring

Although we will be unable to determine if there are hospitalizations or ED visits for opioid withdrawal in patients of clinicians in the intervention group, we will monitor this potential safety issue by comparing clinicians who received a letter to controls to determine if there are any dramatic reductions (>20% from baseline) in opioid prescribing in the post-intervention period as a result of the letter. The U.S. does not have an integrated healthcare system with complete data on hospitalizations for all patients to evaluate induced withdrawal. However, it is possible for us to evaluate if providers induce withdrawal on patients by exhibiting high rates of MME reductions.

8. INTERVENTION DISCONTINUATION

It is not possible to discontinue intervention because prescribers will receive one instance of the informative communication. In the secondary research design, prescribers will receive a follow up communication if the researchers find another case where an affiliated patient has deceased.

9. STATISTICAL CONSIDERATIONS

9.1 General Design Issues

The study is a decedent-cluster randomized field experiment; clusters of prescribers within each decedent were randomly allocated to either treatment or control. This design avoided treatment contamination—clinicians prescribing to the same patient were able to share information about the intervention without diluting its effect. If two or more decedents had the same prescriber, that prescriber was assigned to one and only one of those decedents by random draw. Two factors served as random strata: 1) whether the decedent received a prescription from a clinician with a single or multiple deaths, and 2) whether opioids only, opioids in combination with benzodiazepines, or benzodiazepines only were the cause of death. The first strata is designed to form equivalent groups on prescriber risk posture. The second strata equated preference for type of scheduled drug prescribed in practice. Strata were crossed to form 2 x 3 = 6 randomization groups.

9.2 Sample Size and Randomization

We calculated the sample size needed to detect an effect of the letter intervention using standard formulas for power analysis in cluster randomized trials and the ‘clusterPower’ package in R statistical computing language.(19) For this calculation we used data on decedents and prescribers at the San Diego County Medical Examiner between January 1 and December 31, 2013 published in Lev et al. 2016.(20) We assume a two-tailed test with a 5% Type I error rate

and an 80% chance to detect an effect. Lev et al.(20) found mean of 4.5 prescribers for each decedent in San Diego County. Coefficient of variation was not reported by Lev et al. 2016. We assume it to be 1.22, which implies that 99% of decedents had 20 or fewer prescribers in the year before their death. We assume a 5% difference in means and a standard deviation of +140 daily MME within cluster. Most clinician clusters for process measures have intracluster correlations that fall between 0.05 and 0.15.(21) We assume a more conservative intracluster correlation of 0.2; in our design we randomized by decedent to reduce intracluster correlation. We utilized the Taylor method for calculating variance inflation due to unequal cluster sizes. Under these assumptions, we would need 65 decedents per study arm.

9.2.1 Treatment Assignment Procedures.

The study is a decedent-cluster randomized field experiment; clusters of prescribers within each decedent were randomly allocated to either treatment or control. This design avoided treatment contamination—clinicians prescribing to the same patient were able to share information about the intervention without diluting its effect. If two or more decedents had the same prescriber, that prescriber was assigned to one and only one of those decedents by random draw. Two factors serve as random strata: 1) whether the decedent received a prescription from a clinician with a single or multiple deaths, and 2) whether opioids only, opioids in combination with benzodiazepines, or benzodiazepines only were the cause of death. The first strata is designed to form equivalent groups on prescriber risk posture. The second strata equated preference for type of scheduled drug prescribed in practice. Strata were crossed to form 2 x 3 = 6 randomization groups.

For randomization, six decedent lists were generated from the crossed strata levels. Using random.org's sequence generator, true random integer sequences derived from atmospheric noise determined decedent order in each list. For each ordered list, prescribers to decedents in the first half were those who received the intervention.

9.3 Interim analyses and Stopping Rules

Not applicable

9.4 Outcomes

9.4.1 **Primary outcome**

Our primary analysis assesses changes over time before and after the intervention in natural log-transformed MME between treatment and control.

9.4.2 **Secondary outcomes**

As secondary hypotheses, we evaluate if the intervention cohort had 1) fewer high-dose opioid prescriptions (≥ 50 or > 90 MME per day), or 2) fewer new patients who were started on opioids. High-dose prescriptions were evaluated using censored regression on percent of clinician fills on high-dose. Chance of a fill being a "new start" were evaluated using logistic regression. These analyses were also conducted within a difference-in-differences framework.

9.5 Data Analyses

Descriptive and inferential statistics will be carried out in STATA (Version 14.0; Stata Corporation, College Station, TX). The *cmp* command in STATA will be used to compute a difference-in-differences estimator within a mixed-model censored linear regression.(*17*) The difference-in-differences estimator compares the average change over time in MME dispensed for prescribers in the intervention group, compared to the average change over time for prescribers in the control group. The natural log transformation of MME ensures normally distributed data. Censored regression has a continuous component and a discrete one. Natural log MME doses estimated over days where opioids were dispensed in the name of a prescriber represent the continuous part, and days with no opioids filled in that prescriber's name represent the discrete part of the model. We denote estimation of the dependent variable as $log(\widehat{MME})*$ to distinguish it from uncensored estimation. The analysis is represented by:

$$log(\widehat{MME})*_{ijk} = \beta_1 x_{1ij} + \beta_2 x_{2ij} + \beta_3 x_{3ij} + \delta_{i(k)} \qquad [1]$$

where β_1, $\beta_2$and β_3 are fixed effects coefficients on time, x_1, intervention, x_2,and time by intervention interaction, x_3, respectively. Alphabetic subscripts describe the *i*th prescriber, *j*th prescription filled and *k*th decedent, the nested random intercept $\delta_{i(k)}$is normally distributed with mean zero and variance, $\sigma^2_{i(k)}$, for each *i* prescriber nested in (i.e., having prescribed to) decedent *k*, *i*(*k*). With natural log transformed data, the value 100·[*exp*{β_3} - 1] measures the percentage change in MME attributable to the intervention when data are uncensored.(*18*) However, when data are censored the coefficient β_3 must be adjusted to reflect the effect on the observed outcome. We will evaluate data 3 months pre-intervention and 3 months post-intervention, with

post-intervention beginning one month after the letter was sent to washout contamination by prescriptions written before receipt of the letter but filled afterwards. For the one-year analysis we will include a regressor to control for the 3-month effect. We will perform sensitivity analysis on regression assumptions. As secondary hypotheses, we evaluate if the intervention cohort had 1) fewer high-dose opioid prescriptions (≥ 50 or > 90 MME per day), or 2) fewer new patients who were started on opioids. High-dose prescriptions will be evaluated using censored regression on percent of clinician fills on high-dose. Chance of a fill being a "new start" will be evaluated using logistic regression. These analyses will also be conducted within a difference-in-differences framework.

10. DATA COLLECTIO N AND QUALITY ASSURANCE

10.1 Data Collection Forms

The Controlled Substance Utilization Review and Evaluation System (CURES) will provide records of opioids dispensed at California pharmacies attributable to each provider in our sample treating all civilian, non-Veteran's Administration and non-institutionalized patients. CURES is California's prescription drug monitoring program. The Medical Examiner has authority to use CURES for the purpose of educating practitioners and others in lieu of disciplinary, civil, or criminal actions, in accordance with the California State's Health and Safety Code § 11165(c)(2). First name, last name, date of birth and address identified each decedent in the medical examiner reports and CURES data. Drug Enforcement Agency (DEA) number identified the prescribers in CURES data. Data from eligible clinicians will be extracted from CURES and kept onsite at the medical examiner's office for de-identified analytic file preparation. A file stripped of patient and clinician identifiers will be prepared and released for analysis on secure servers at the University of Southern California.

10.2 Data Management

Each of the participating sites will create an extract from the CURES database for all deceased patients where prescription drug over-dosage was the primary cause or contributed to the cause of death. These records will be accessed, extracted, then transferred to the localized database in the coordinating center on a weekly basis. The coordinating center has created programs and quality control queries for transforming all of the data into a standard model (Observational Medical Outcomes Partnership Common Data Model, version 3). The data collection forms will be CURES database extracts.

10.3 Quality Assurance

10.3.1 Training

All project personnel handling study data will be certified by the Collaborative IRB Training Initiative (CITI) program, which consists of courses in the Protection of Human Research Subjects for Biomedical Research. Staff will trained on the permissible values present in the CURES database, frequency of update, and expected volumes of data.

10.3.2 Quality Control Committee

The quality control committee consisted of practicing clinicians from each participating clinical organization. They reviewed automatically refreshing dashboards for potential deviations in coding systems and appropriate values for codes included in the outcome measures. These dashboards were reviewed prior to each email distribution.

10.3.3 Metrics

Quality control metrics were based on reports were based on verifications for prescription with National Drug Codes for Schedule II/III/IV drugs following the FDA Drug Schedule.

10.3.4 Protocol Deviations

Our task tracking system, JIRA was used to track and document issues. Each issue included an assignee and a reviewer.

10.3.5 Monitoring

In addition to data quality reviews, we will also review the integrity of the interventions. It is the primary role of the data analysts to: 1) determine the appropriate number of eligible providers to reach sufficient statistical power, 2) record provider information in a secure, password protected database located at LA County Medical Examiner-Coroner's office and, 3) track enrolled providers in regards to their intervention status and progress. On an approximately quarterly basis, staff would visit headquarters of participating sites and verify functionality of decision support tools. Additionally, practicing clinicians on our study team had the ability to monitor electronic medical record interventions in their own health systems.

11. <u>PARTICIPANT RIGHTS AND CONFIDENTIALITY</u>

11.1 Institutional Review Board (IRB) Review

The study protocol will be reviewed and approved by the University of Southern California's Institutional Review Board (IRB). Individual site protocols will also be submitted for review and approval by the California site local IRB.

11.2 Informed Consent Forms

We will request a waiver of consent.

11.3 Participant Confidentiality

Data will be recorded on a localized data warehouse. Data will be kept in encrypted files on computers in locked offices at San Diego County Medical Examiner's Office facilities. Only study investigators will have password protected access to a restricted patient and prescriber information.

11.4 Study Discontinuation

Following each DSMB meeting, the board will make recommendations to the local IRBs as to whether the study should continue or if changes to the protocol are necessary for continuation.

12. <u>COMMITTEES</u>

Not applicable

13. <u>PUBLICATION OF RESEARCH FINDINGS</u>

Publication of results from our research will follow the NIH Public Access Policy, which requires that we submit to the National Library of Medicine's PubMed Central an electronic version of final, peer-reviewed manuscripts upon acceptance for publication, to be made publicly available no later than 12 months after the official date of publication.

14. REFERENCES

D. Boudreau et al., “Trends in long-term opioid therapy for chronic non-cancer pain,” Pharmacoepidemiol. Drug Saf., vol. 18, no. 12, pp. 1166–1175, 2009.

D. Dowell, T. M. Haegerich, and R. Chou, “CDC Guideline for Prescribing Opioids for Chronic Pain - United States, 2016,” MMWR Recomm. Rep., vol. 65, no. 1, pp. 1–49, Mar. 2016.

C. S. Florence, C. Zhou, F. Luo, and L. Xu, “The Economic Burden of Prescription Opioid Overdose, Abuse, and Dependence in the United States, 2013,” Med. Care, vol. 54, no. 10, pp. 901–906, Oct. 2016.

G. P. Guy Jr et al., “Vital Signs: Changes in Opioid Prescribing in the United States, 2006-2015,” MMWR Morb. Mortal. Wkly. Rep., vol. 66, no. 26, pp. 697–704, Jul. 2017.

D. N. Juurlink, “Rethinking ‘doing well’ on chronic opioid therapy,” CMAJ, vol. 189, no. 39, pp. E1222–E1223, Oct. 2017.

E. E. Krebs et al., “Effect of Opioid vs Nonopioid Medications on Pain-Related Function in Patients With Chronic Back Pain or Hip or Knee Osteoarthritis Pain: The SPACE Randomized Clinical Trial,” JAMA, vol. 319, no. 9, pp. 872–882, Mar. 2018.

K. Kroenke et al., “Challenges with Implementing the Centers for Disease Control and Prevention Opioid Guideline: A Consensus Panel Report,” Pain Med., vol. 20, no. 4, pp. 724–735, Apr. 2019.

L. Scholl, “Drug and Opioid-Involved Overdose Deaths — United States, 2013–2017,” MMWR Morb. Mortal. Wkly. Rep., vol. 67, 2019, doi: 10.15585/mmwr.mm6751521e1.

15. SUPPLEMENTS/APPENDICES

OPIOIDS LIST

**Included**	**Excluded**
Codeine	Buprenorphine
Fentanyl tablet	Butorphanol

Fentanyl patch	Dihydrocodeine
Hydrocodone	Fentanyl lozenge
Hydromorphone	Fentanyl powder*
Methadone	Fentanyl spray*
Morphine	Levorphanol tartrate
Oxycodone	Meperidine
Oxymorphone	Opium
Tapentadol	Tramadol

*Excluded because prescription was likely administered in office by a healthcare professional

List taken from CDC Commonly Prescribed Opioids

https://www.cdc.gov/mmwr/volumes/65/rr/rr6501e1.htm
