# Supplementary material for: Effect of Prescriber Notifications of Patient’s Fatal Overdose on Opioid Prescribing at 4 to 12 Months: A Randomized Clinical Trial
Source: JAMA Netw Open. 2023 Jan 6;6(1):e2249877. doi: 10.1001/jamanetworkopen.2022.49877 (PMC9856831; doi:10.1001/jamanetworkopen.2022.49877)
Supplement: Supplement 2. — Data Sharing Statement [file jamanetwopen-e2249877-s002.pdf]

Data Sharing Statement

Doctor. Effect of Prescriber Notifications of Patient's Fatal Overdose on Opioid Prescribing at 4 to 12 Months. *JAMA Netw Open*. Published January 06, 2023.
doi:10.1001/jamanetworkopen.2022.49877

Data
Data available: No

Additional Information
Explanation for why data not available: Data was obtained from the Department of Justice of California. Per our agreement with them we cannot release the data ourselves.
